# MRGPRX2-mediated mast cell response to drugs used in perioperative procedures and anaesthesia

**DOI:** 10.1038/s41598-018-29965-8

**Published:** 2018-08-02

**Authors:** Arnau Navinés-Ferrer, Eva Serrano-Candelas, Alberto Lafuente, Rosa Muñoz-Cano, Margarita Martín, Gabriel Gastaminza

**Affiliations:** 1 Biochemistry Unit, Biomedicine Department, Faculty of Medicine, University of Barcelona, Casanova 143, Barcelona, 08036 Spain; 2 Laboratory of Clinical and Experimental Respiratory Immunoallergy, IDIBAPS, Barcelona, Spain; 3 Department of Anaesthesiology, Clínica Universidad de Navarra, Pamplona, Spain; 4 Allergy Section, Neumology Department, Hospital Clinic, University of Barcelona, Barcelona, Spain; 5 Department of Allergy and Clinical Immunology, Clínica Universidad de Navarra, Pamplona, Spain

## Abstract

The study of anaphylactoid reactions during perioperative procedures and anaesthesia represents a diagnostic challenge for allergists, as many drugs are administered simultaneously, and approximately half of them trigger allergic reactions without a verifiable IgE-mediated mechanism. Recently, mast cell receptor MRGPRX2 has been identified as a cause of pseudo-allergic drug reactions. In this study, we analyse the ability of certain drugs used during perioperative procedures and anaesthesia to induce MRGPRX2-dependent degranulation in human mast cells and sera from patients who experienced an anaphylactoid reaction during the perioperative procedure. Using a β-hexosaminidase release assay, several drugs were seen to cause mast cell degranulation *in vitro* in comparison with unstimulated cells, but only morphine, vancomycin and cisatracurium specifically triggered this receptor, as assessed by the release of β-hexosaminidase in the control versus the MRGPRX2-silenced cells. The same outcome was seen when measuring degranulation based on the percentage of CD63 expression at identical doses. Unlike that of the healthy controls, the sera of patients who had experienced an anaphylactoid reaction induced mast-cell degranulation. The degranulation ability of these sera decreased when MRGPRX2 was silenced. In conclusion, MRGPRX2 is a candidate for consideration in non-IgE-mediated allergic reactions to some perioperative drugs, reinforcing its role in mast cell responses and their pathophysiology.

## Introduction

Allergic reactions occurring during perioperative procedures and anaesthesia may be severe and life-threatening for the patient. The study of these reactions represents a diagnostic challenge for allergists, as many drugs are administered simultaneously, including anaesthesia inducers, opiates, muscle relaxants, antibiotics, nonsteroidal anti-inflammatory drugs (NSAIDs), iodinated contrast agents, plasma expanders or dyes. In fact, a major issue in these reactions is that the triggering drug cannot be identified in approximately half of cases, since the allergy study is negative^1^.

Study protocols establish that, when skin tests with suspected drugs have yielded negative results, a challenge or dose-provocative test (DPT) must be carried out to rule out an allergy^2^. However, this should not be performed, for example, with neuromuscular blocking agents (NMBAs), such as atracurium or succinylcholine, and other anaesthetic drugs^3^. This type of test also entails a high risk, particularly in patients who have experienced anaphylaxis. Therefore, there is a clear need to deepen our understanding on the mechanism of action of some of these reactions.

Recently, the Mas-Related G-Protein-coupled Receptor X2 (MRGPRX2) has been identified as a target for certain drugs, such as neuromuscular blocking agents (atracurium, rocuronium) or fluoroquinolones (ciprofloxacin, levofloxacin), associated with systemic pseudoallergic or anaphylactoid reactions^4,5^. This receptor is expressed on human mast cells, the main cells involved in anaphylactic reactions.

In this paper we will analyse whether MRGPRX2 may be involved in pseudoallergic reactions associated with drugs used in anaesthesia, in which an IgE-mediated mechanism is not identified.

To this end, we intend to test drugs used in perioperative procedures and anaesthesia, such as opiates, muscle relaxants, iodinated contrast agents, antibiotics and NSAIDs, based on MRGPRX2 expression in a mast cell line, and to analyse the ability of these drugs to induce a response mediated by this receptor.

We hypothesize that the MRGPRX2 receptor may be responsible for allergic reactions occurring during anaesthesia. To confirm this hypothesis, we tested drugs capable of degranulating mast cells in cells where the expression of MRGPRX2 had been selectively silenced, to determine the role of the receptor in such process. Moreover, sera from both patients who had suffered an allergic reaction during anaesthesia and healthy controls were also tested to assess the reactivity in our cell model.

## Results

### Analysis of the ability of drugs used during perioperative procedures and anaesthesia to induce degranulation in human mast cells

**Figure 1:**
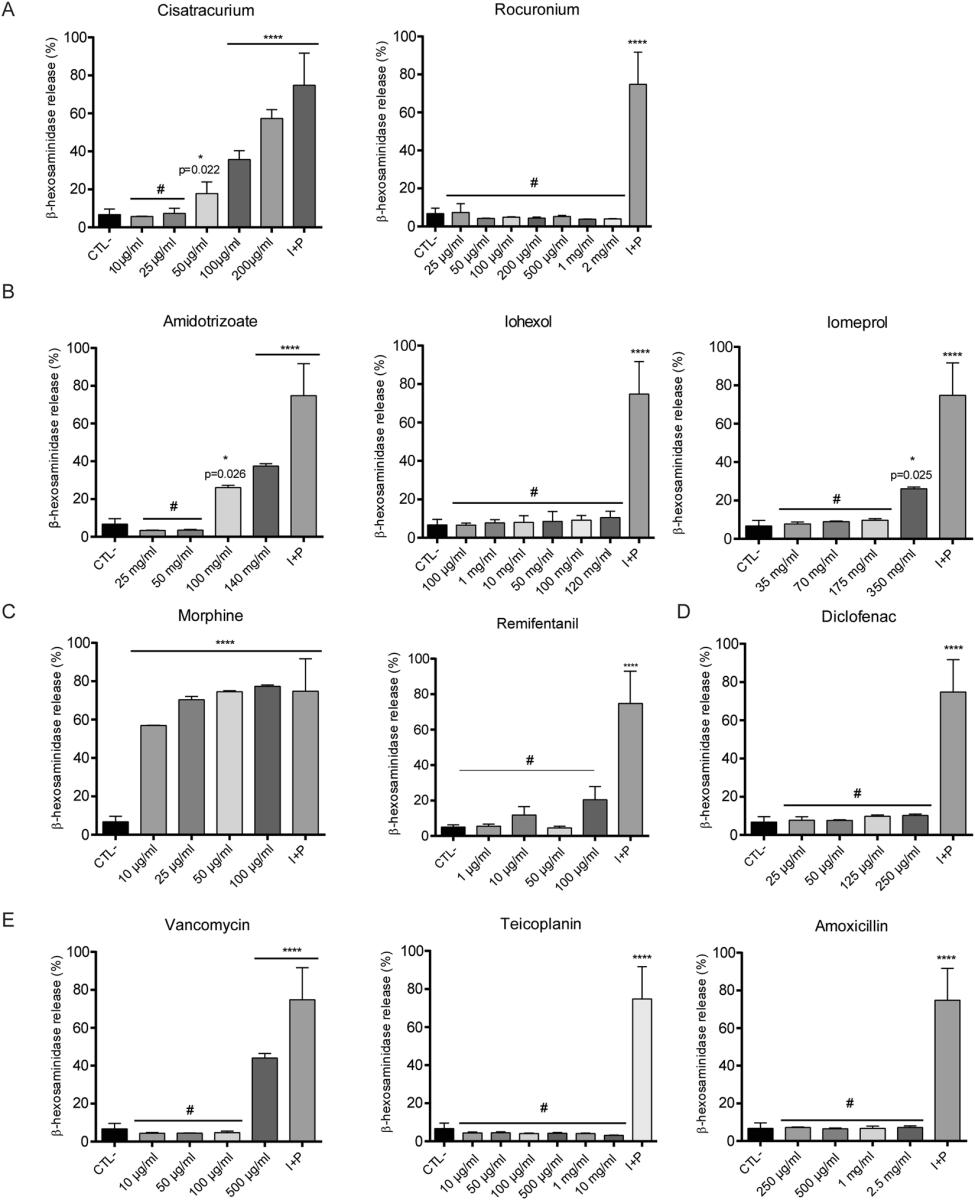
Determination of mast cell degranulation by NMBAs, iodinated contrasts, opiates, non-steroidal anti-inflamatory diclofenac and antibiotics. β-hexosaminidase assays of several NMBAs (**A**) iodinated contrasts (**B**) the opiates morphine and remifentanil (**C**) the non-steroidal anti-inflammatory diclofenac (**D**) and several antibiotics (**E**) on LAD2 mast cells. Bars show the mean ± SEM of at least 3 replicates of the experiment. First bar (CTL−; negative control) correspond to unstimulated cells and last bar (I + P; positive control) correspond to cells stimulated with Ionomycin and PMA. Statistical significance (^#^p > 0.99; *p < 0.05; ****p < 0.0001; unpaired ANOVA with Bonferroni post-hoc test) is relative to CTL−.

We first tested the ability of several drugs used in perioperative procedures and anaesthesia (cisatracurium, rocuronium, meglumine amidotrizoate, iohexol, iomeprol, propofol, vancomycin, teicoplanin, amoxicillin-clavulanic acid, diclofenac, remifentanil and morphine) to directly stimulate mast cells. To this end, we incubated LAD2 mast cells with different concentrations of these drugs and analysed their degranulation response using a β-hexosaminidase activity assay. Unstimulated cells (CTL-) were used in all cases as negative controls to evaluate basal degranulation, and cells stimulated with phorbol 12-myristate 13-acetate (PMA) plus ionomycin (I + P) were used as positive controls for degranulation. Our data showed that, among the NMBAs tested, only cisatracurium, at doses over 50 µg/mL, was able to induce cell degranulation (Fig. 1A). Of the three iodinated contrasts used (Fig. 1B), meglumine amidotrizoate (at doses over 100 mg/mL) and iomeprol (at doses over 350 mg/mL), but not iohexol, induced cell degranulation. Opiate morphine (but not remifentanil) induced cell degranulation at very low doses (10 μg/mL) (Fig. 1C), whereas, of all antibiotics tested, only vancomycin at a dose of 500 μg/mL induced cell degranulation (Fig. 1E). As an exception, the ability of propofol to induce mast cell degranulation was determined by flow cytometry, because the colour of the compound interfered with the β-hexosaminidase colorimetric assay. Propofol did not induce mast cell degranulation (data not shown). In short, cisatracurium (17.75 ± 2.03, p = 0.022), meglumine amidotrizoate (26 ± 0.73, p = 0.026), iomeprol (26.12 ± 0.52, p-value = 0.025), morphine (56.92 ± 0.14, p < 0.0001) and vancomycin (44.06 ± 1.39, p < 0.0001) caused mast cell degranulation *in vitro* when examined in comparison with unstimulated cells (6.71 ± 0.62) (Table 1).

**Table 1:** Drugs tested to induce mast cell degranulation.

			Doses used in Prick’s test	Minimal doses to induce degranulation	Maximum ineffective dose	Mean (% B-hex release or % CD63 expression)	p-value
Tested by β-hexosaminidase release	Unstimulated cells		N/A	N/A	N/A	6.71 ± 0.62	
	Muscle relaxants	atracurium	100 µg/ml	50 µg/ml	—	17.75 ± 2.03	**0**.**022**
		rocuronium	10 µg/ml	—	2 mg/ml	3.97 ± 0.066	>0.999
	Iodinated contrast agents	meglumine amidotrizoate	90 mg/ml	100 mg/ml	—	26 ± 0.73	**0**.**026**
		iohexol	2.4 mg/ml	—	120 mg/ml	10.58 ± 1.92	>0.999
		iomeprol	70 mg/ml	350 mg/ml	—	26.12 ± 0.52	**0**.**025**
	Opiates	remifentanil	50 ug/mL	—	100 µg/mL	20.46 ± 7.39	0.995
		morphine	50 µg/ml	10 µg/ml	—	56.92 ± 0.14	<**0**.**0001**
	non-steroidal anti-inflamatory	diclofenac	2.5 mg/ml	—	250 µg/ml	10.18 ± 0.46	>0.999
	Antibiotics	vancomycin	50 mg/ml	500 µg/ml	—	44.06 ± 1.39	<**0**.**0001**
		teicoplanin	100 mg/ml	—	10 mg/ml	3.113 ± 0.12	>0.999
		amoxicillin-clavulanic	2.5 mg/ml	—	2.5 mg/ml	7.25 ± 0.47	>0.999
Tested by CD63 expression in cell membrane	Unstimulated cells		N/A	N/A	N/A	55.26 ± 2.15	
	General anesthesics	propofol	1 mg/ml	—	100 µg/ml	63.51 ± 2	0.17

The corresponding skin test doses, the minimal dose to induce degranulation and the maximum ineffective dose of each drug are shown. The mean value of the percentage of β-hexosaminidase release ± SEM, or the percentage of CD63 expression ± SEM are shown with the statistical significance (p-value) of each test in comparison with unstimulated cells.

Cell viability was measured in all cases in order to discard degranulation due to cell mortality. Cell viability was similar in all cases and over 90% (data not shown).

### Mast cell degranulation by cisatracurium, morphine and vancomycin depends on MRGPRX2 expression

**Figure 2:**
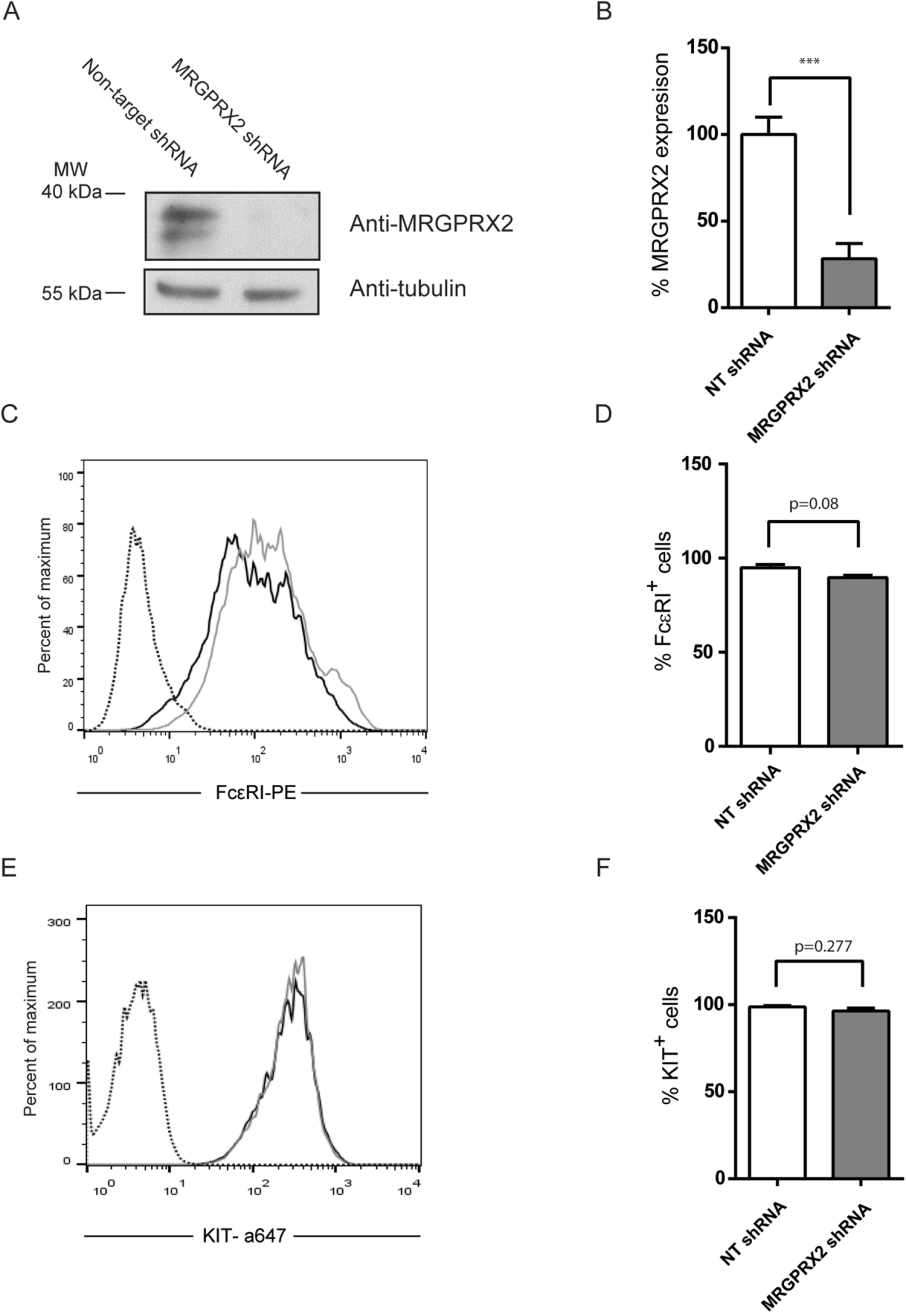
Determination of the expression of the MRGPRX2 receptor. (**A**) Western blot of mast cells silenced for MRGPRX2 (MRGPRX2-shRNA) or control cells (Non-target-shRNA). Percentage of MRGPRX2 expression on MRGPRX2-shRNA or NT-shRNA cells (Non-target-shRNA). Data is the mean of six experiments. (**B**) FcεRI (**C**) and KIT (**E**). Dot lines correspond to isotype control, grey lines correspond to Non-target shRNA cells and black lines correspond to MRGPRX2-shRNA cells. Positive cells from a representative experiment are indicated in the histogram. Bar charts represent the percentage of FcεRI (**D**, n = 3) and KIT (**F**, n = 4) positive cells. Data show the mean ± SEM. Statistical significance (***p < 0.001) was determined using unpaired t-test with Welch’s correction and it is relative to non-target shRNA.

To test whether the stimulation of mast cells by these drugs is mediated by the MRGPRX2 receptor, we obtained human mast cells with reduced MRGPRX2 receptor expression from the LAD2 line, using a lentiviral knockdown system. Cells transduced with the scramble sequence, which we designated as non-target shRNA, were used as controls and analysed together with the silenced cells (MRGPRX2-shRNA). Receptor silencing was confirmed by Western blot and flow cytometry (Fig. 2A,B). FcεRI and KIT, the most characteristic receptors involved in mast cell degranulation and cell survival, were analysed following the MRGPRX2 knockdown. As shown in Fig. 2C–F, the levels of FcεRI (94.9 ± 1.75 vs. 89.73 ± 1.24, p = 0.08) and KIT (98.68 ± 0.71 vs. 96.28 ± 1.77, p = 0.277) expression were similar in both the control and MRGPRX2-silenced cells.

**Figure 3:**
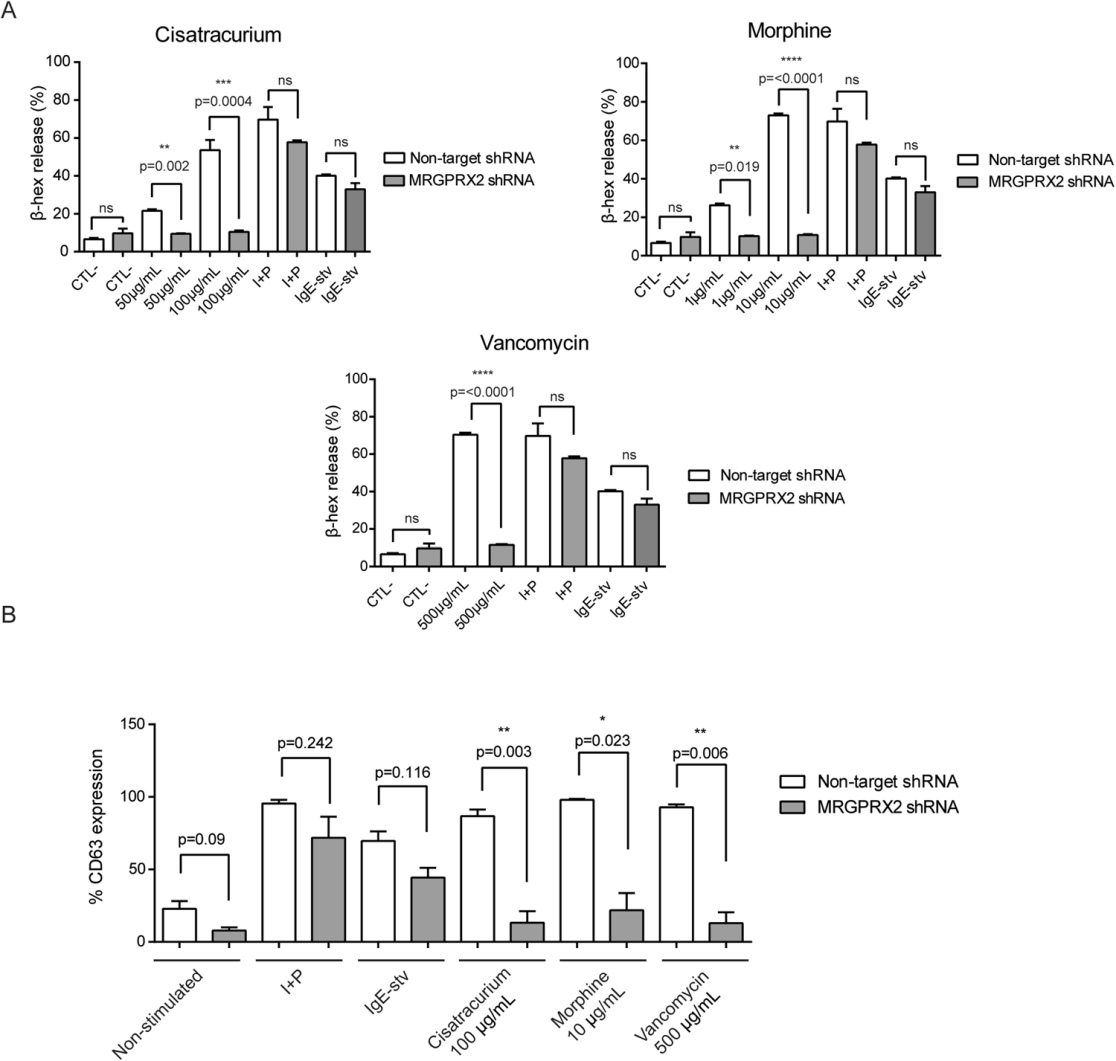
Morphine, vancomycin and cisatracurium responses are mediated through MRGPRX2. (**A**) β-hexosaminidase assays and (**B**) percentage of CD63 expression in Non-target and MRGPRX2-silenced cells tested with Cistracurium (50–100 µg/mL), Morphine (1–10 µg/mL) or Vancomycin (500 µg/mL). Data show the mean ± SEM. Statistical significance (*p < 0.05, **p < 0.01, ****p < 0.0001; unpaired t-test with Welch’s correction) is for non-target shRNA versus MRGPRX2 shRNA.

We then analysed the ability of cisatracurium, morphine and vancomycin to induce cell degranulation based on MRGPRX2 knockdown expression. We ruled out meglumine amidotrizoate and iomeprol because such response was only seen at very high doses exceeding the concentration of drugs usually administered to patients for iodinated contrast agents^6^ (Table 1). Our results showed that MRGPRX2-silenced mast cells had a reduced degranulation response compared to the non-silenced cells (non-target), as measured by β-hexosaminidase release following their activation with morphine at 1 µg/mL (10.11 ± 0.29 vs. 26.17 ± 0.96, p = 0.0019) and 10 µg/mL (10.72 ± 0.44 vs. 72.95 ± 0.1, p < 0.0001), vancomycin at 500 µg/mL (11.41 ± 0.43 vs. 70.32 ± 1.08, p < 0.0001), and cisatracurium at 50 µg/mL (9.45 ± 0.27 vs. 21.59 ± 0.80, p = 0.002) and 100 µg/mL (10.41 ± 0.76 vs. 53.63 ± 5.33, p = 0.0004) (Fig. 3). No significant differences were observed between control, crosslinked IgE or I + P-stimulated cells (CTL-: 6.61 ± 0.69 vs. 9.67 ± 2.47, p = 0.280; I + P: 69.74 ± 6.65 vs. 57.78 ± 1, p = 0.133, IgE-stv: 40.07 ± 0.6723 vs. 32.89 ± 3.258 p = 0.0564) (Fig. 3A).

Flow cytometry was used to confirm the degranulation response based on the percentage of CD63 expression on the cell membrane. We observed a similar reduced response in MRGPRX2-silenced mast cells for cisatracurium at 100 µg/mL (13.21 ± 8.02 vs. 86.63 ± 4.74, p = 0.003), morphine at 10 µg/mL (21.83 ± 11.83 vs. 97.90 ± 0.72, p = 0.023) and vancomycin at 500 µg/mL (12.93 ± 7.53 vs. 98.2 ± 2.06, p = 0.006). For the most part, cells maintained their ability to respond to I + P (I + P: 95.47 ± 2.46 vs. 71.8 ± 14.47, p = 0.242) and crosslinked IgE (IgE-streptavidin: 69.55 ± 6.65 vs. 44.35 ± 6.65, p = 0.116). Unstimulated cells also showed no differences (22.83 ± 5.34 vs. 7.78 ± 2.34, p = 0.09) (Fig. 3B). These results demonstrate that the drugs mainly induced MRGPRX2-mediated degranulation.

### Sera from patients who experienced anaphylactoid reactions induce MRGPRX2-mediated mast cell degranulation

The main problem related to the study of anaphylactoid reactions was the great diversity of drugs that can be administered simultaneously to the same patient during the anaesthetic procedure. Hence, in addition to exposing mast cells separately to different drugs, we decided to analyse the response of our mast cell model to sera collected from patients who had suffered an anaphylactoid reaction during anaesthesia.

For this purpose, we obtained several serum samples following the onset of an allergic reaction to perioperative drugs (from time of reaction to 24 h), with a negative skin test (skin prick and intradermal tests). Three of the samples were collected further than 24 hours from the onset of the reaction. Five serum samples from healthy donors (control sera) were also collected. Three of them were from patients after anaesthesia procedure with several drugs showing no adverse reaction. The tryptase and histamine levels of all the allergic patients were also determined (Table 2).

**Table 2:** Sera from patients who had suffered an allergic reaction to one or more drugs during anesthesia.

Drugs	Serum code	Time of collection	Reaction	Tryptase levels (µg/L) (Basal vs. Reaction)	Histamine levels (ng/mL)
MORPHINE (negative skin tests)	P1	0 h–24 h	Urticaria after the administration of MORPHINE with negative skin tests.	6.19–7.21	48.33
	P2	0 h–24 h	Generalized exanthema following the administration of MORPHINE with negative skin tests.	Unknown-2.98	17.31
REMIFENTANIL (negative skin tests)	P3	0 h–24 h	Generalized exanthema, angioedema, sneezing, dyspnea, tachycardia, hypertension, following the administration of REMIFENTANIL with negative skin tests.	Unknown-1.52	40.78
ROCURONIUM (negative skin tests)	P4	0 h–24 h	Generalized exanthema and hypotension after the administration of ROCURONIUM with negative skin tests.	4.78–58.8	48.82
CISATRACURIUM (negative skin tests)	P5	0 h–24 h	Trunk exanthema following the administration of CISATRACURIUM with negative skin tests.	3.17–3.3	32.21
ATRACURIUM (negative skin tests)	P6	0 h–24 h 1 month	Generalized exanthema following the administration of ATRACURIUM with negative skin tests.	1.71–3.12	81.29
SEVERAL DRUGS ADMINISTERED DURING INDUCTION OF ANESTHESIA (negative skin tests)	P7	0 h–24 h	Generalized exanthema during induction of anesthesia (propofol, succinylcholine and fentanyl) with negative skin tests.	Unknown-2.72	27.36
	P8	0 h–24 h	Generalized exanthema during induction of anesthesia (diazepam, fentanyl and propofol) with negative skin tests.	2.33–2.69	24.26
	P9	0 h–24 h	Generalized exanthema during induction of anesthesia (multiple drugs) with negative skin tests.	3.08–3.06	23.25
	P10	0 h–24 h	Bronchospasm, wheezing, general exanthema during induction of anesthesia (propofol, atracurium, diazepam and fentanyl) with negative skin tests.	1.96–1.94	33.43
	P11	0 h–24 h	Wheezing, bronchospasm and exanthema during induction of anesthesia (etomidate, succinylcholine, fentanyl and diazepam) with negative skin tests.	3.33–6.04	59.76
	P12	0 h–24 h 48 h	Hypotension and tachycardia during induction of anesthesia (propofol, diazepam, remifentanil, atracurium, amoxicillin-clavulanic acid) with negative skin prick tests.	<1.0–<1.0	223.29
	P13	0 h–24 h 2 months	Severe hypotension, bronchospasm and general exanthema during induction of anesthesia (propofol, succinylcholine and fentanyl) with negative skin prick tests.	3.15–5.13	119.85
CONTROLS	C1-C2	—	Healthy subjects	N/D	N/D
	C3*	0 h	Healthy subject who received atracurium, rocuronium, fentanyl, remifentanil, morphine, propofol, paracetamol, and ondasetron.	N/D	N/D
	C4*	0 h	Healthy subject who received atracurium, diazepam, fentanyl, morphine, propofol and succinylcholine.	N/D	N/D
	C5*	0 h	Healthy subject who received atropine, sugammadex, rocuronium, fentanyl metamizol, morphine and propofol.	N/D	N/D

The tryptase levels (µg/L) are shown as basal levels versus levels at the time of anaphylactoid reaction. Histamine levels were determined at the time of anaphylactoid reaction, considering <28 ng/mL as the basal levels for a healthy individual. N.D. means not determined.

**Figure 4:**
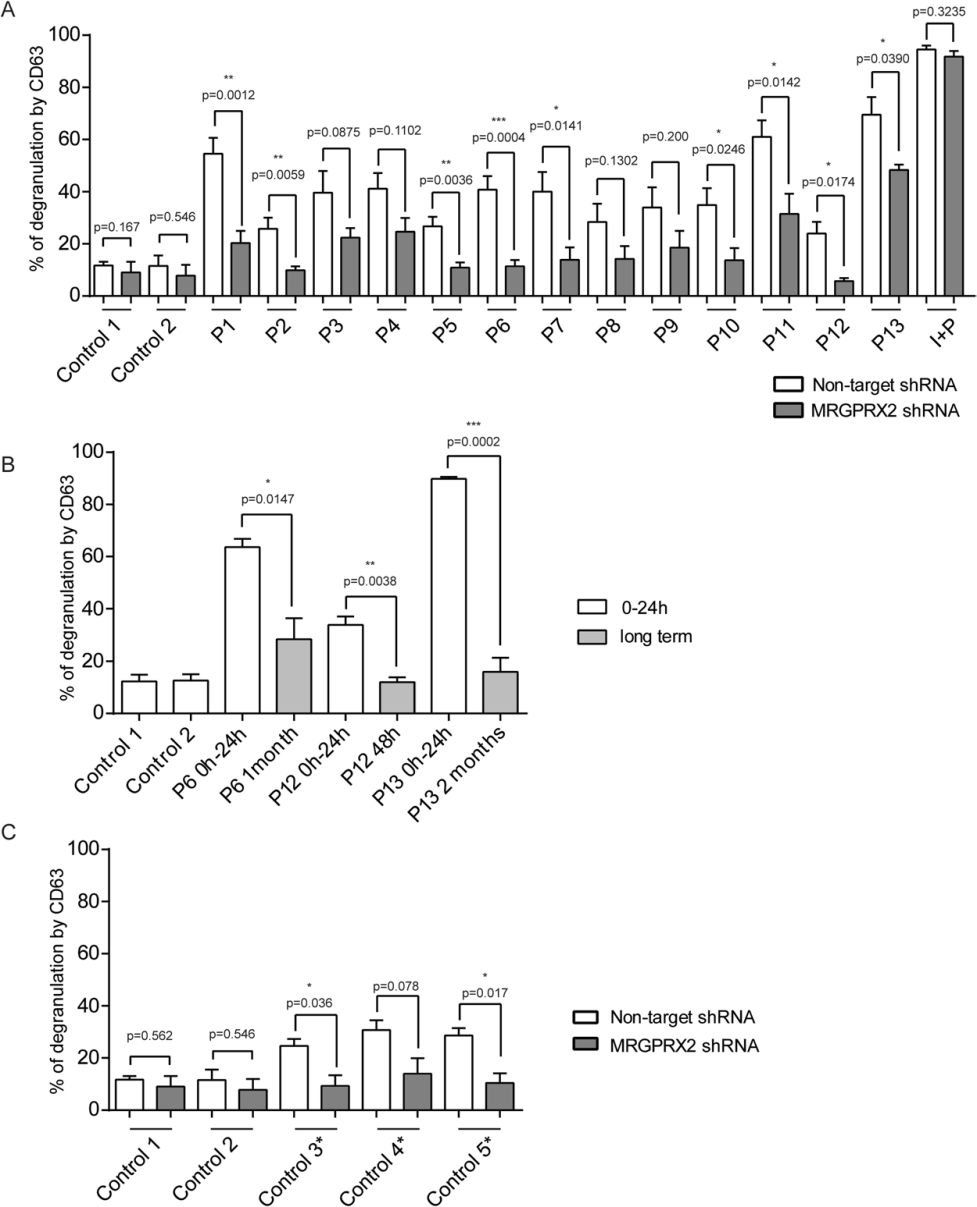
Skin test negative sera from patients are capable of inducing a degranulation response in mast cells. This degranulation capacity is reduced when MRGPRX2 is downregulated. (**A**) Percentage of CD63 expression of non-target or MRGPRX2 knockdown mast cells incubated with sera from healthy controls (control sera) or sera from patients (see Table 2). Data show the mean ± SEM. Statistical significance (*p < 0.05, **p < 0.01, ****p < 0.0001; unpaired t-test with Welch’s correction) is for non-target shRNA versus MRGPRX2 shRNA. (**B**) Percentage of CD63 expression in mast cells incubated with control sera or patient’s sera at different time points (0 h to 24 h versus long term collection). Data show the mean ± SEM. Statistical significance (*p < 0.05, **p < 0.01, ****p < 0.0001; unpaired t-test with Welch’s correction) is for 0–24 h versus long term collection. (**C**) Percentage of CD63 expression of non-target or MRGPRX2 knockdown mast cells incubated with sera from healthy controls and healthy patients who received several drugs. Data show the mean ± SEM. Statistical significance (*p < 0.05, **p < 0.01, ****p < 0.0001; unpaired t-test with Welch’s correction) is for non-target shRNA versus MRGPRX2 shRNA. All data is representative of three independent experiments.

First, we assessed the ability of the sera to induce degranulation in mast cells by measuring CD63 expression using flow cytometry. Unlike the control sera (13.78 ± 2.26 and 11.58 ± 3.964), most of the patient sera were activators to some extent. After MRGPRX2 knockdown, we observed a statistically significant reduction in the activation capacity of most of the sera except for sera from patients P3, P4, P8 and P9. The control sera showed no significant differences (Fig. 4A). This indicated that the degranulation ability of mast cells was partly dependent on MRGPRX2 receptor expression.

We collected sera from some of our patients at long term time points. As shown in Fig. 4B, all tested sera collected after 24 hours of the time of reaction showed statistically significant less capability to activate mast cell degranulation.

Finally, we analysed the sera from patients who went through a perioperative procedure without any anaphylactoid response (C3*, C4*, C5*). We observed that these sera had some ability to induce mast cell degranulation compared to control sera from healthy patients (C1 and C2). This activity was reduced in MRGPRX2-silenced cells (Fig. 4C).

## Discussion

The newly discovered MRGPRX2 receptor is a non-canonical G-protein-coupled receptor expressed on human mast cells that plays a role in host defence and allergic inflammation^4^. Recent findings suggest a role for this receptor in non-IgE-mediated drug-induced pseudoallergic reactions^5^. *In vitro* studies have demonstrated that this receptor triggers a different type of mast cell degranulation process than the IgE-dependent one. The substance P-dependent activation of the MRGPRX2 receptor induces a quick, and almost immediate, secretion of small and relatively spherical granules. On the contrary, FcεRI-dependent degranulation results in a more gradual degranulation, with longer and heterogeneous granules. *In vivo*, the allergic reaction caused by MRGPRX2 is a faster and more localized reaction compared to the more intense, prolonged and systemic reaction triggered by the FcεRI receptor^7^. In clinical practise, Mertes *et al*. described that IgE-mediated anaphylaxis are more prone to increase tryptase levels and induce bronchospasm and cardiovascular symptoms while non-IgE mediated reactions frequently show isolated cutaneous symptoms without an increase in tryptase^8^.

The purpose of our research was to study the recurring clinical issue of allergic reactions to drugs used during perioperative procedures and anaesthesia, which in many cases may be severe or life-threatening for the patient. Despite their low prevalence, we observed that the frequency of these reactions was higher than expected, up to 1 in every 385 procedures^9^. We focussed in drugs which induce adverse reactions without a verifiable IgE-mediated mechanism.

Among the drugs analysed in this study, morphine and vancomycin resulted in mast cell degranulation at administered doses. Both drugs triggered mast cell degranulation through MRGPRX2 since silencing of the receptor significantly reduced degranulation. This result falls in line with the ability of these two drugs to induce non-IgE-mediated reactions according to clinical experience^10^. Skin tests are usually negative in these cases, and the reaction frequently appears during the first administration of the drug, without the possibility of becoming sensitized in a previous contact. We have not a complete explanation for the negative result of skin tests in these patients. Skin mast cells have MRGPRX2 receptors in their membrane, and one should wait a positive skin test via MRGPRX2. However, MRGPRX2 is a low-affinity receptor (µg/ml range)^11,12^ compared to IgE sensitivity against allergens (ng/ml range)^13,14^. Thus, MRGPRX2 receptor activation *in vivo* might require to be in close proximity to high local concentrations of the drug^10^. Other possible explanation could be the modification of the drug after binding to skin proteins or the production of a metabolite that modifies the capacity of activation of MRGPRX2. On the other hand, we should also take into consideration that, even if IgE-mediated reactions are usually associated with positive skin tests, some authors claim to be cautious with the interpretation of these tests and propose basophil activation tests (BAT) as a more reliable measurement of specific IgE-mediated anaphylaxis^15^. In this context, it has been reported that the results of the prick test with vancomycin does not correlate with the “red man syndrome” (RMS) elicited after intravenous infusion of vancomycin, suggesting that the route of administration could elicit different responses^16^.

The fact that the drug, which did not produce a positive skin test, was able to activate *in vitro* mast cells via MRGPRX2, could be explained by the different local concentration of the drug in a cell culture *in vitro* compared to the *in vivo* situation, where mast cells may be more heterogeneous and scattered in the skin and the drug can suffer modifications in its structure and inactive metabolites may be produced.

As in previous studies carried out with atracurium^5^, cisatracurium was seen to cause a dose-dependent MRGPRX2-mediated degranulation in LAD2 cells. Interestingly, cases of IgE-mediated allergic reactions to atracurium have also been reported, thus suggesting a dual mechanism of action for this drug^17^. We detected no mast cell degranulation in response to rocuronium. In fact, and according to our data, rocuronium proved to significantly induce degranulation mediated by mouse ortholog receptor MrgprB2 rather than by MRGPRX2, thus proving the existence of differences between the human receptor and its mouse ortholog^5^.

Our data showed that all sera collected at the onset of the allergic reaction (<24 h) in patients with a negative skin test to all the suspicious drugs could induce mast cell degranulation. This capacity is not seen in sera collected at a further time from the anaphylactoid reaction (>24 h). Patients who had an anaphylactoid reaction to morphine, cisatracurium and atracurium showed a statistically significant reduction in MRGPRX2-silenced cells compared to non-target cells. Interestingly, patients who had experienced a reaction to remifentanil and rocuronium did not show a significant decrease. This correlates with our data showing that rocuronium and remifentanil did not trigger any mast cell activation *in vitro*. Additionally, sera from patients who received several drugs also showed a statistically significant reduction of the degranulation capacity except for those from patients 8 and 9. Interestingly some of the drugs administered to these latter patients, such as succinylcholine^5^ or fentanyl^18^, have been described in the past as non-histamine releasers^14^.

Consequently, the ability of sera to induce mast cell degranulation could be explained by the residual presence of drugs or drug metabolites in the serum samples, indicating that some compounds found in patients could trigger a mast cell response^4,6,19^. This hypothesis is supported by the fact that the serum from patients who received several drugs without eliciting an allergic response can also induce some mast cell degranulation *in vitro*. But the higher degranulation capacity of the sera from patients who suffered an anaphylactoid reaction could suggest that some allergic compounds released by the immune system at the moment of the reaction may also participate, inducing mast cell degranulation.

It has been widely reported that iodinated contrast mediums (ICMs) amidotrizoate and iomeprol can induce allergic reactions^6^. However, in our model, the doses needed to induce β-hexosaminidase release in mast cells were also affecting cell viability. For that reason, we consider that the detected β-hexosaminidase activity was due to cell mortality, which causes general release of intracellular components, rather than to a degranulation process. The other ICM tested, iohexol, did not activate mast cells *in vitro* at any doses assessed. Therefore, these drugs were excluded from the MRGPRX2 analysis and further studies are required to determine the molecular basis and mechanism of ICM-mediated allergic reactions.

In the near future, it may prove useful to combine drugs and analyse the variable effects of each combination on mast cell degranulation, to collect patient sera at different time points following the anaphylactoid reaction, and to follow up on the kinetics and ability of the sera to induce mast cell degranulation.

In short, our results show that the MRGPRX2 receptor is a potential cause of non-IgE-mediated allergic reactions to several drugs commonly used during perioperative procedures and anaesthesia. Supporting our results, a recent study reported that morphine analogues can induce mast cell degranulation mediated by the MRGPRX2 receptor^10^.

Our research broadens the scope for the study of non-IgE-mediated reactions. Several conserved polymorphisms for the MRGPRX2 receptor have been described, with 1172 being listed at present in the RefSeq database^20^, of which 152 are missense mutations. An analysis of MRGPRX2 receptor polymorphisms in the genome of allergic patients would enable us to determine whether this receptor has genomic variability, which could explain why only some patients experienced exacerbated reactions to certain drugs. This genomic variability could increase the receptor’s affinity to the drugs or induce a stronger intracellular response. One possible explanation for this is the fact that this receptor is differentially expressed in the mast cells of different patients, triggering a greater response in those with increased expression. In this regard, MRGPRX2 has been found to be increased in patients with severe chronic urticaria^21^. The increased expression of this receptor may explain why the injection of neuropeptides, such as substance P, enhances wheal reactions in patients with chronic urticaria as compared to healthy controls.

Overall, we consider that the knowledge about the different forms, variants, or expression levels of the MRGPRX2 receptor may constitute a powerful diagnostic tool for evaluating the predisposition of patients to suffer adverse reactions in response to certain drugs. Such knowledge would allow professionals to personalize the combination of drugs used based on each patient’s genetic profile, so as to reduce the number of anaphylactoid reactions occurring during clinical practice.

## Materials and Methods

This study was approved by the institutional review board of the University of Navarra.

### Biological samples

The LAD2 human mast cell line was kindly provided by Dr. D. Metcalfe, (NIH Washington). The HEK 293LTV cell line (Cell Biolabs Inc, San Diego, CA, USA) was used for lentivirus production. The control sera from healthy patients and the sera from patients who had experienced an allergic reaction in response to a muscle relaxant (rocuronium, cisatracurium or atracurium), an opiate (morphine or remifentanil), or after receiving several drugs during the anaesthesia induction, and who had negative allergy tests to these drugs, were collected at the Clínica Universidad de Navarra (Table 2). These patients had already been included in previous studies^1,9^. All serum samples, were extracted during the onset of the allergic reaction, no later than 24 h. We also obtained 3 samples from our patients at a further time from the reaction (48 h, 1 and 2 months). Control sera from healthy individuals and from patients that underwent anaesthesia procedure with no anaphylactoid reaction were also collected. All of them are indicated in Table 2.

### Ethics statement

The study was approved by the State Ethics Committee and the Ethics Research Committee of the University of Navarra. The authors performed these procedures in accordance with the approved guidelines, obtaining informed consent from each subject before conducting the experiments.

### Drugs used in this study

We incubated mast cells separately with different drugs and analysed their degranulation response. Our study included several drugs which had frequently been seen to cause non-IgE-mediated immediate allergic drug reactions, including opiates (morphine from B. Braun at a dose of 10 mg/mL and remifentanil from Kern Pharma 1 mg powder), muscle relaxants (cisatracurium from Pfizer at 2 mg/mL; rocuronium from Merck Sharp Dohme at 10 mg/mL), general anaesthetics (propofol-lipuro from Braun at 10 mg/mL), NSAIDs (sodium diclofenac from Novartis at 75 mg), iodinated contrast agents (iohexol from GE Healthcare at 240 mg/mL; iomeprol from Bracco at 714.4 mg/mL; and meglumine amidotrizoate from Juste Laboratories at 280 mg/mL), and antibiotics (amoxicillin-clavulanic acid from Sandoz at 1000 mg/200 mg; vancomycin from Normon at 500 mg; and teicoplanin from Sanofi at 400 mg). We included amoxicillin-clavulanic acid as a negative control because its associated allergic reactions have often been described as IgE-dependent.

### Lentivirus production and MRGPRX2 silencing

We used MRGPRX2 receptor MISSION shRNA plasmid DNA MAS-related GPR, member X2 (Sigma. St Louis. MO, USA), which had been validated previously^22^. The lentiviruses were produced by co-transfection with lipofectamine of the MRGPRX2-silencing sequence cloned in the pLKO.1 plasmid and the plasmids encoding the lentiviral capsid in HEK 293LTV cells. Controls were performed with a control sequence (scramble) that had proved to be harmless in protein-silencing eukaryotic cells. We proceeded as described by Ainsua-Enrich *et al*.^23^. Mast cell-positive clones were selected with puromycin (1 µg/mL), and silencing was confirmed by western blot (anti-MRGPRX2 purified mouse polyclonal antibody B02P, Abnova, Germany; and anti-α-tubulin [DM1A clone] Sigma [Sigma, St. Louis, MO, USA]) and flow cytometry (PE anti-human MRGPRX2 clone K125H4 Biolegend, San Diego, CA, USA) following the procedure described elsewhere^24^. FcεRI expression was analysed using mouse anti-human FcεRI-PE (eBioscience, San Diego, CA, USA), while KIT expression was evaluated using mouse anti-cKIT (Ab81 clone, Santa Cruz Biotechnology, Inc. Santa Cruz, CA, USA) and a secondary goat anti-mouse IgG antibody, Alexafluor 647 (Invitrogen Life Technologies, Carlsbad, CA, USA), using FACSCalibur flow cytometry (FACScan; BD Biosciences, Mountain View, CA, USA).

### Mast cell degranulation assays

Degranulation was analyzed based on the levels of β-hexosaminidase activity at the supernatant or on CD63 expression on the cell membrane, assessed by flow cytometry, as described in previous studies^23,25^. β-hexosaminidase is an enzyme found inside mast cell granules and released at the supernatant after cell degranulation. CD63 is a protein found in the granules and expressed on the cell membrane after degranulation. For each type of essay, we briefly incubated 2 × 10^4^–1 × 10^5^ cells at 37 °C for 30 minutes with several concentrations of the drugs described earlier. For IgE-stimulation, we sensitized mast cells overnight with biotinylated IgE (Abbiotec, San Diego, CA, USA) (0.1 µg/mL), and stimulated them for 30 minutes at 37 °C with streptavidin (0.4 µg/mL) to induce IgE crosslinking. As a positive control for degranulation, we incubated the samples with ionomycin (200 µg/mL) and PMA (10 ng/mL) for 30 minutes at 37 °C.

To carry out the degranulation assays with patient sera, we incubated each serum sample with 1 × 10^5^ mast cells in Tyrode’s buffer for 30 minutes at 37 °C, at a ratio of 1:1, for a total volume of 100 µL. Because of the colour of the serum samples, which interfered with the β-hexosaminidase colorimetric test, only flow cytometry assays with the CD63-APC marker were performed for the sera.

To rule out the drugs’ toxic effects on the cells, a viability count was also carried out in all cases, using trypan blue in the β-hexosaminidase assays and propidium iodide (PI) in the flow cytometry assays.

### Statistical analysis of the results

All study data are shown as a mean value ± SEM. Multiple group comparisons were performed using one-way analysis of variance (ANOVA), followed by the Bonferroni post-hoc test. Student’s T-test with Welch’s correction was used to conduct all analyses between both groups (patients and controls). In addition, the analyses were carried out using GraphPad Prism 6. P-values < 0.05 (two-tailed) were considered statistically significant. The β-hexosaminidase assays were performed in triplicate, and all experiments were carried out at least three times. The CD63 analyses carried out by flow cytometry were also performed at least three times.

### Data availability

The datasets generated during and/or analysed during the current study are available from the corresponding author on reasonable request.
