# Author Correction: MRGPRX2-mediated mast cell response to drugs used in perioperative procedures and anaesthesia

**DOI:** 10.1038/s41598-021-91844-6

**Published:** 2021-06-08

**Authors:** Arnau Navinés-Ferrer, Eva Serrano-Candelas, Alberto Lafuente, Rosa Muñoz-Cano, Margarita Martín, Gabriel Gastaminza

**Affiliations:** 1 Biochemistry Unit, Biomedicine Department, Faculty of Medicine, University of Barcelona, Casanova 143, 08036 Barcelona, Spain; 2 Laboratory of Clinical and Experimental Respiratory Immunoallergy, IDIBAPS, Barcelona, Spain; 3 Department of Anaesthesiology, Clínica Universidad de Navarra, Pamplona, Spain; 4 Allergy Section, Neumology Department, Hospital Clinic, University of Barcelona, Barcelona, Spain; 5 Department of Allergy and Clinical Immunology, Clínica Universidad de Navarra, Pamplona, Spain

Correction to: *Scientific Reports* https://doi.org/10.1038/s41598-018-29965-8, published online 02 August 2018

The original version of this Article contained errors in the Reference list. The authors omitted the below paper, which is listed as Reference 22.

Kirshenbaum AS, Akin C, Wu Y, Rottem M, Goff JP, Beaven MA, Rao VK, Metcalfe DD. Characterization of novel stem cell factor responsive human mast cell lines LAD 1 and 2 established from a patient with mast cell sarcoma/leukemia; activation following aggregation of FcepsilonRI or FcgammaRI. Leuk Res. 2003 Aug;27(8):677–82.

As a result, in the Materials and Methods section,

“The LAD2 human mast cell line was kindly provided by Dr. D. Metcalfe, (NIH Washington).”

now reads:

“The LAD2 human mast cell line was kindly provided by Dr. D. Metcalfe, (NIH Washington)^22^.”

Consequently, References 23–26 were incorrectly listed as References 22–25.

The original Article has been corrected.

